# A Part-Based Probabilistic Model for Object Detection with Occlusion

**DOI:** 10.1371/journal.pone.0084624

**Published:** 2014-01-17

**Authors:** Chunhui Zhang, Jun Zhang, Heng Zhao, Jimin Liang

**Affiliations:** School of Life Science and Technology, Xidian University, Xi'an, Shaanxi, China; Instituto de Fisica Interdisciplinar y Sistemas Complejos IFISC (CSIC-UIB), Spain

## Abstract

The part-based method has been a fast rising framework for object detection. It is attracting more and more attention for its detection precision and partial robustness to the occlusion. However, little research has been focused on the problem of occlusion overlapping of the part regions, which can reduce the performance of the system. This paper proposes a part-based probabilistic model and the corresponding inference algorithm for the problem of the part occlusion. The model is based on the Bayesian theory integrally and aims to be robust to the large occlusion. In the stage of the model construction, all of the parts constitute the vertex set of a fully connected graph, and a binary variable is assigned to each part to indicate its occlusion status. In addition, we introduce a penalty term to regularize the argument space of the objective function. Thus, the part detection is formulated as an optimization problem, which is divided into two alternative procedures: the outer inference and the inner inference. A stochastic tentative method is employed in the outer inference to determine the occlusion status for each part. In the inner inference, the gradient descent algorithm is employed to find the optimal positions of the parts, in term of the current occlusion status. Experiments were carried out on the Caltech database. The results demonstrated that the proposed method achieves a strong robustness to the occlusion.

## Introduction

**Figure 1:**
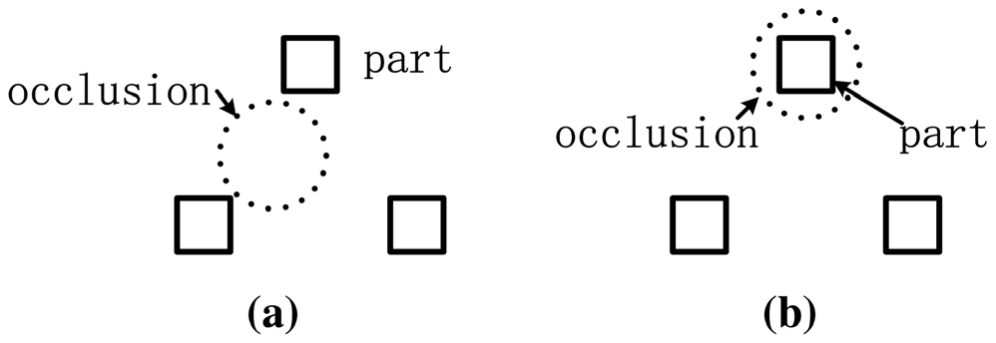
Two kinds of occlusions faced by the part-based model. (a) Occlusion which does not overlap the part areas. (b) Occlusion overlapping the part areas.

Object detection [1] is a classical problem in the field of computer vision. Among the numerous methods for object detection, the statistical-based approaches [2]–[13] have become mainstream. They discriminate the given object from others by learning it, hence achieving a more robust detection. As a typical statistical-based method, the part-based model has attracted increasing attention in the past decade [5]–[11], [14]–[19]. As the name implies, the part is the local area of the object. The part-based models can capture both the local appearance and spatial structural information of the object simultaneously, which makes these methods robust to the variations of the object pose and appearance to some extent. Similar with other statistical-based methods, the part-based methods also include two fundamental problems: training and detection. The former refers to the modeling of the part appearance and spatial relationship among the parts. The latter refers to the optimization problem for acquiring the information about the parts (such as their position and occlusion status). It is worthy to emphasize that, most part-based methods pay attention to the part areas only. Therefore, they are robust only to the occlusions which do not overlap the part areas (as illustrated in Figure 1.(a)). However, if the occlusions overlap the part areas, as illustrated in Figure 1.(b), not only would the occluded parts be influenced, the non-occluded ones would also be shifted from their right positions due to the spatial relationship among the parts (refer to the experiments below for more details).

### Related work and our contributions

**Figure 2:**
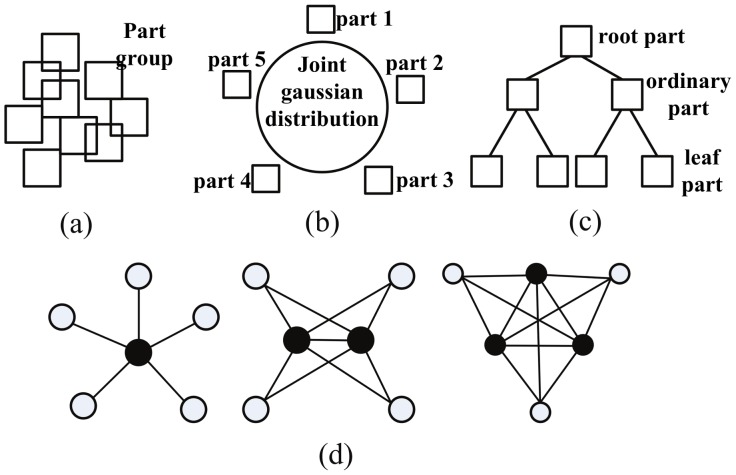
Spatial relationship among parts. (a) Bag model. (b) Constellation model. (c) Pictorial model. (d) 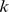-fan model (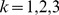 from left to right).

In the past years, many part-based methods have emerged, such as the bag model [6], [7], constellation model [8], [15], pictorial structure model [9], [16], star model [10], [14], vocabulary based method [17], [18] and 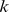-fan model [11]. The spatial relationship among their parts is illustrated in Figure 2.

However, The bag model almost consider none of spatial relationship among parts. The other part-based models improve the performance of detection by adding the spatial relationship among the parts. Especially, the pictorial structure model and the 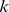-fan model perform fast detection via dynamic programming [20], [21], since the appearance of generalized distance transform (GDT) [22] greatly reduces the time complexity of the dynamic programming. However, they does not allow cycles in the spatial relationship [20]. There are many solutions for this problem [23]–[27], of which the simplest technique is the gradient descent algorithm (GD).

**Figure 3:**
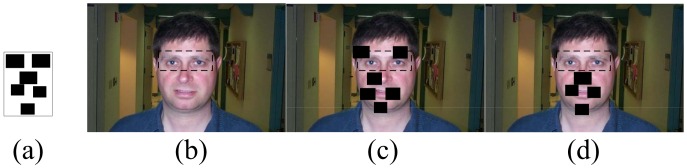
Influence of the disabled parts on the detection of the normal ones. (a) is the manual label of the face. In (b), the eyes have been occluded (the occlusion is represented by the dotted box). (c) shows the detection results that the disabled parts degrade the detection of the normal ones. (d) illustrates the detection results after the occluded parts are discarded from the model.

Although the above part-based models belong to the category of sparse representation, they still suffer from the problem of shading the part areas (as demonstrated in Figure 3). In this paper, these shaded parts are named disabled parts. some methods [19] employed a kind of part appearance representation which is robust to the occlusion, but the robustness is limited, especially when the occlusion region is large. In the literature about the occlusion, ignoring the occluded parts from the model is the most intuitive idea to solve the occlusion problem [8], [14], [28], [29]. In general, it can be achieved by estimating a mask for the test image. However, the mask variable is difficult to model in the objective function to achieve a precise inference and cannot work in the case of large occlusion. Papandreou et al. proposed to solve the occlusion problem by using a robust objective function [30], which weakens the role of occlusion parts, but it is difficult to completely eliminate the influence of the occluded areas. Li et al. [31] solved the occlusion problem under a novel RANSAC framework. However, it is difficult to incorporate the spatial relationship to improve the detection of the keypoints. In [32], [33], the authors solved the occlusion problem under the sparse framework, which is usually used in the case of batch image processing.

This paper considers applying the part-based method in object detection, with special emphasis on occlusion handling, and propose a part-based probabilistic model with an alternative detection scheme. In order to increase the detection accuracy, we constructed a fully connected graph to describe the spatial relationship among parts in the stage of training. Each edge is represented by a 2D Gaussian distribution for the vector difference of the position coordinates. Moreover, we introduced a penalty term to the objective function ensure us to obtain a more accurate detection result. For the occlusion problem, we assigned a binary status variable to each part to indicate whether it is occluded or not, and proposed a method to model the prior probability of the occlusion status variable. Then, according to the Bayesian theory, we constructed a new posterior probability as the objective function. In the stage of detection, We designed two alternative procedures, which are named as the inner inference and outer inference. The former used the GD to determine the positions of the parts given the current occlusion status variable. The outer inference is responsible for determining the occlusion status of the parts according to their current positions. To address the detection efficiently, we adopted a stochastic tentative method in the outer inference. In addition, in the procedure of the detection, we incorporated the validity test mechanism to avoid the invalid inner inference results.

## Methods

Consider a model with 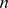 parts 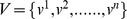. A detection result of a given image is expressed as 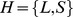. The argument 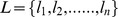 is the position variable, where 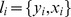 denotes the position of part 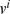. The argument 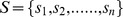 represents the occlusion status variable, where 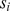 is a Boolean variable (if part 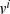 is a normal part, 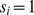; otherwise, 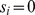 for the disabled part). We define the object function as
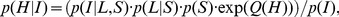
(1)which is the posterior probability of a result 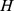 given a test image 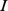, where 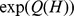 is a penalty term and 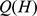 is defined in the following subsection.

### Construction of the model

In Eq. 1, the posterior probability contains four items. 1. 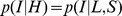, which represents the total matching probability of the normal parts. 2. 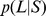 represents the *priori* probability of a spatial relationship among normal parts. 3. 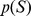, a priori probability of the occlusion. 4. The penalty term 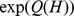.

The total matching probability of all of the normal parts is
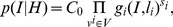
(2)where 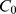 is a constant for a test image 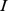, 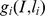 is the matching probability of a single part [11].

For a *priori* probability of the spatial relationship 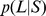, we employed the fully connected graph to represent the spatial relationship among the parts. However, as demonstrated in Figure 3.(c), the disabled parts will severely affect the detection of the normal parts because of the edges between them. We overcame this problem by discarding the edges connected to the disabled parts. In addition, the positions of the disabled parts were supposed to follow the independent uniform distribution [8]. Therefore,
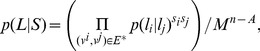
(3)where 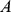 is the number of the normal parts with 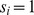, 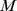 is a constant representing the number of possible positions where a part could be placed, 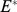 is the edge set of a fully connected graph, and 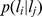 is defined as 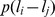 which follows the 2D Gaussian distribution.

Let 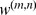 denote the conditional probability of part 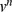 being shaded under the condition that 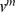 is disabled. If the mean distance 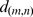 between 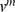 and 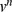 satisfies that 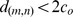
(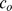 is a constant), we have proved that (please refer to the *Appendix S1* for the deduction.)
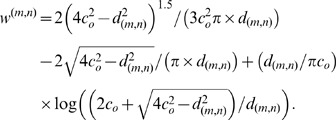
(4)Then a *priori* probability 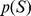 is calculated as
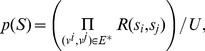
(5)where
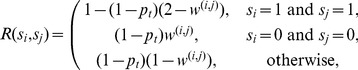
(6)represents the joint probability of the occlusion status about the part pair 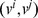. Where 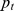 is a constant standing for the probability of a part being present, and 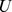 is a normalization constant.

The penalty term 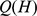 aims at improving the detection results by emphasizing the weak parts, and also for regularizing the argument space of the objective function. It is defined as
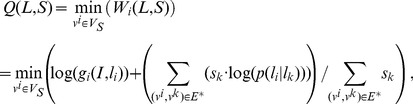
(7)where 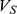 is the part set in which the occlusion status of each element is 1.

Substitute Eq. 2, 3, 5, 7 into Eq. 1, the posterior probability can be rewritten as
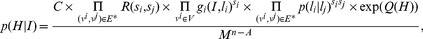
(8)where 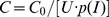 is a constant for a test image 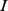. Applying the logarithm and minus operations to both sides of Eq. 8, we have
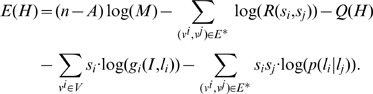
(9)The above expression is the right objective function for detection.

### Detection

In the step of the detection, we look for an optimal detection result 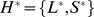 with minimum energy, which is
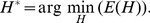
(10)In this paper, we adopted the strategy of alternative optimization to solve the above problem. The basic idea is to let 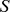 search in the space of 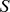 (called the outer inference), and after each movement of 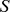, the inner inference searches the current optimal part positions 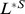. These two procedures are performed alternately until the terminal conditions are satisfied.

#### Inner inference and Outer inference

In the inner inference, given the current status vector 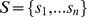, Eq. 9 can be expressed as
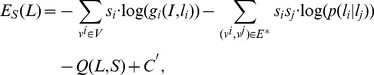
(11)where 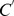 is a constant. Eq 11 is the right objective function in inner inference. we used the gradient descent algorithm (GD) to search the current optimal position variable for each part 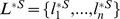.

In the outer inference, given 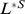, the object function Eq. 9 becomes the function depending on 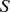 only:
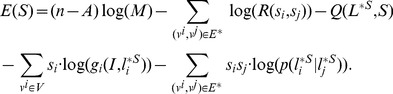
(12)


As aforementioned, the outer inference is responsible for determining the occlusion status variable. At the beginning of the outer inference, the occlusion status variable is assumed to be 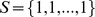, implying that no occlusion happens to any part. The aim of the outer inference is to find the next probable 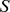 to reduce the value of Eq. 12.

**Figure 4:**
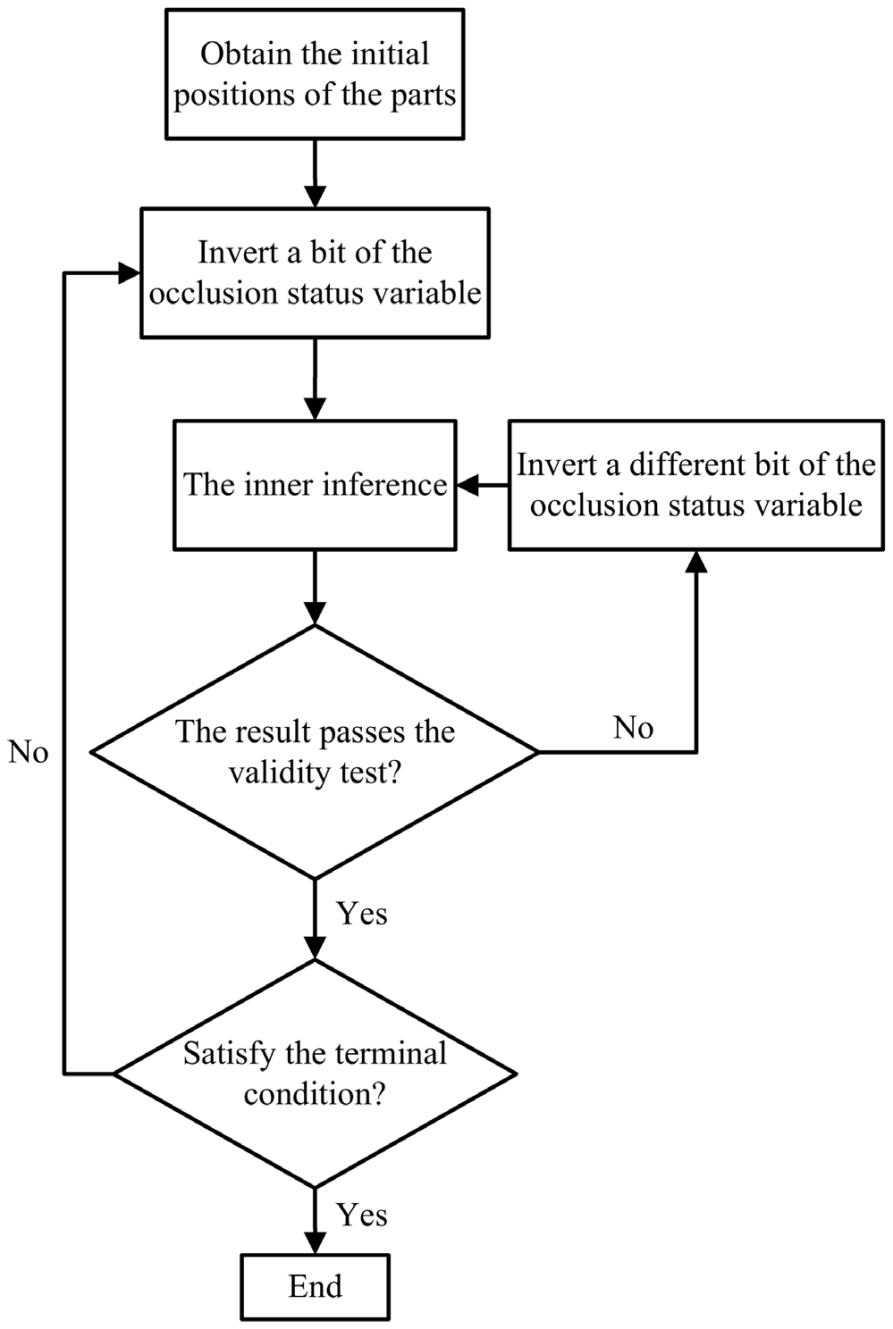
Flow chart of the outer inference.

Due to the discreteness of the 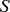 space, we adopted a stochastic tentative method to address the outer inference. In each iteration, we calculated the gradient vector 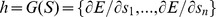 for Eq. 12. If 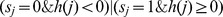 holds, we consider the 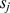 as a feasible descending bit, and consider the 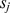 which has the minimal value of 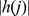 as the most irresolute bit. The procedure of the outer inference is illustrated in Figure 4 and detailed in Table 1.

**Table 1:** Algorithm 1: Outer inference.

**Figure 5:**
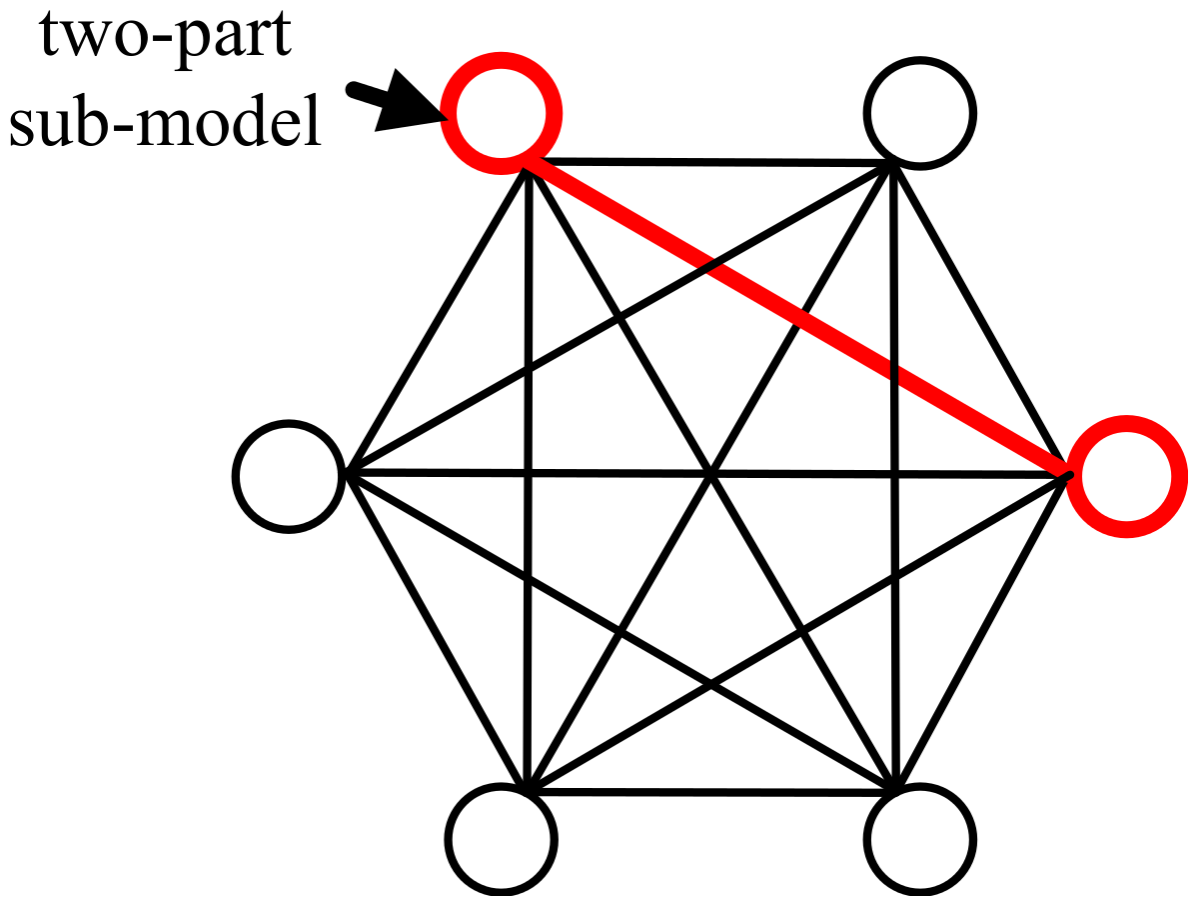
Two-part sub-model.

**Input:** The initial occlusion status variable 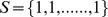 , the initial energy value 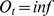 ;
Step 1. Use the simulated annealing algorithm to obtain the initial position, then obtain current optimal position 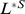 via the inner inference, if it passes the validity test (explained below), go to Step 3, otherwise go to Step 2;
Step 2. Use all of the two-part sub-model (illustrated in Figure 5) to make an inner inference (determine the positions of these two parts), until a two-part sub-model 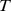 whose results can pass the validity test appear. Then, estimate the approximate positions of other parts except for the two parts in 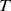 , and obtain their position by GD further. If none of the two-part models can pass the validity test, quit the outer inference in failure;
Step 3. **While** the result 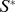 is not altered and the maximum iteration number 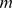 is not reached,
(a) Calculate gradient vector 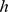 ;
(b) If 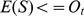 , 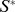 is updated as 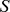 , and 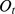 is updated as 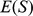 ;
(c) In 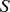 , search the feasible descending bits;
(d) If there is no feasible descending bit, invert the most irresolute bit in 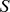 , and go to Step 3g, otherwise go to Step 3e;
(e) If there is only one feasible descending bit, invert it, and go to Step 3g, else go to Step 3f;
(f) If there are at least two feasible descent bits, therein invert the corresponding bit with a probability proportional to its gradient absolute value;
(g) Carry out the GD algorithm for 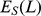 , if the results cannot pass the validity test, go to Step 3h;
(h) Choose a different bit to invert again randomly, go to Step 3g;
**End While**;
**Output:** The status result 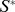 and 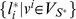 .

The validity test is used to validate whether the inner inference has obtained a feasible result. For a two-part model, if the likehood
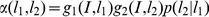
(13)is larger than some threshold, this two-part model is defined to pass the validity test. A full-part model is defined to pass the validity test if there is at least one two-part sub-model passing the validity test. Step 3g to Step 3h is the procedure of inner inference, and can avoid the solutions from deviating from the right occlusion status variable. Finally, after we have obtained the output, we could estimate the position of the disabled parts just by the spatial relationship among all of the parts, i.e. minimizing the following expression:
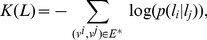
(14)where 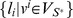 is the known variable, which has been obtained in Algorithm 1 (Table 1).

## Results and Discussion

In this section, we tested the performance of our method on the Faces dataset in the Caltech database [8], [34], and the performance of 1-fan [11] is compared. For this dataset, as done in [11], six parts were selected: the left eye, the right eye, nose, the left corner of the mouth, the right corner of the mouth and the chin (defined as the part 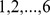 respectively). In our experiment, the distance error 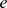 is defined as the mean distance of 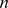 parts from the detection results to its corresponding ground truth. The smaller 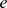 is, the better the given model performs on this specific test image.

**Figure 6:**
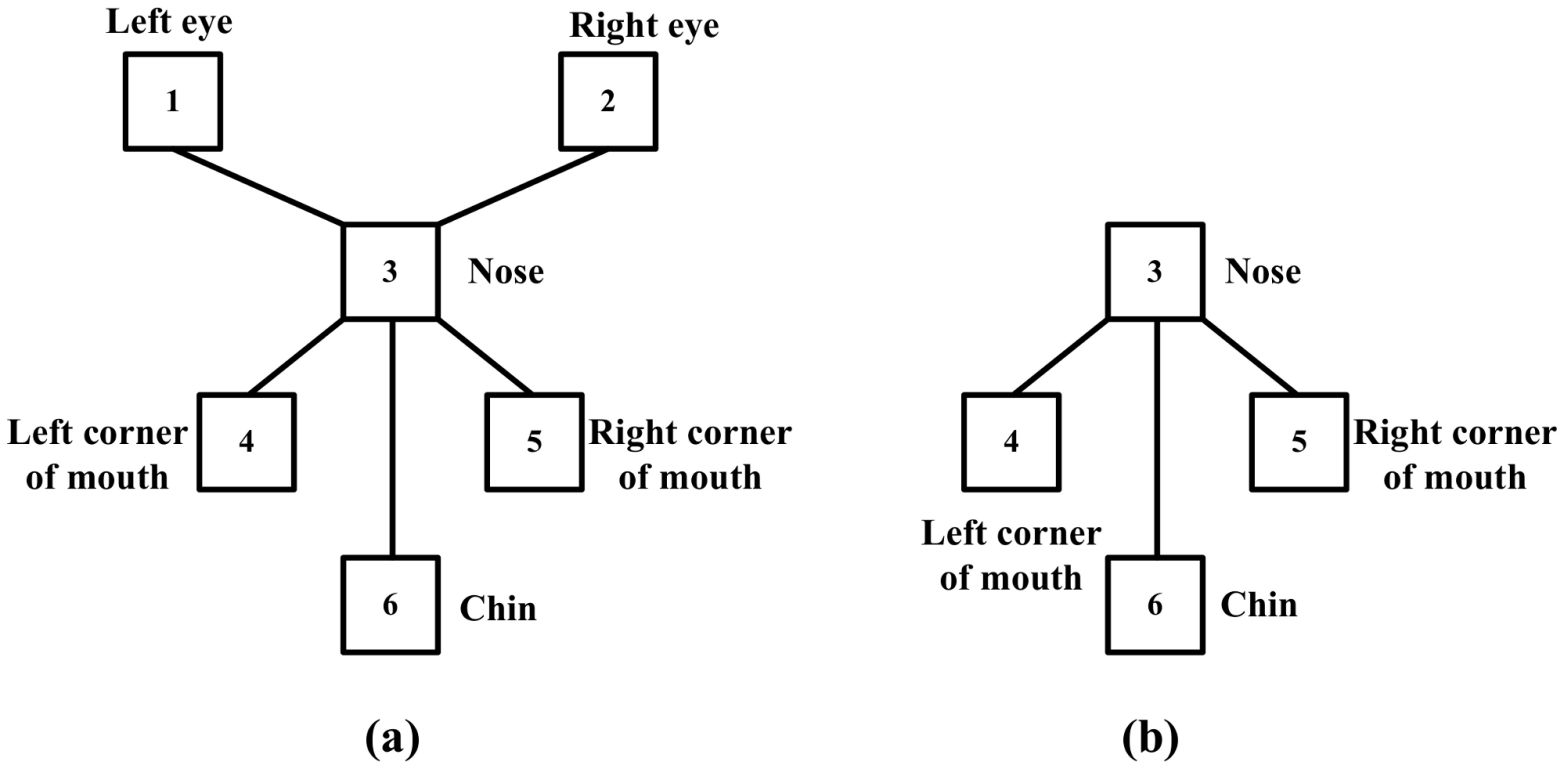
1-fan model and discarded 1-fan model.

**Figure 7:**
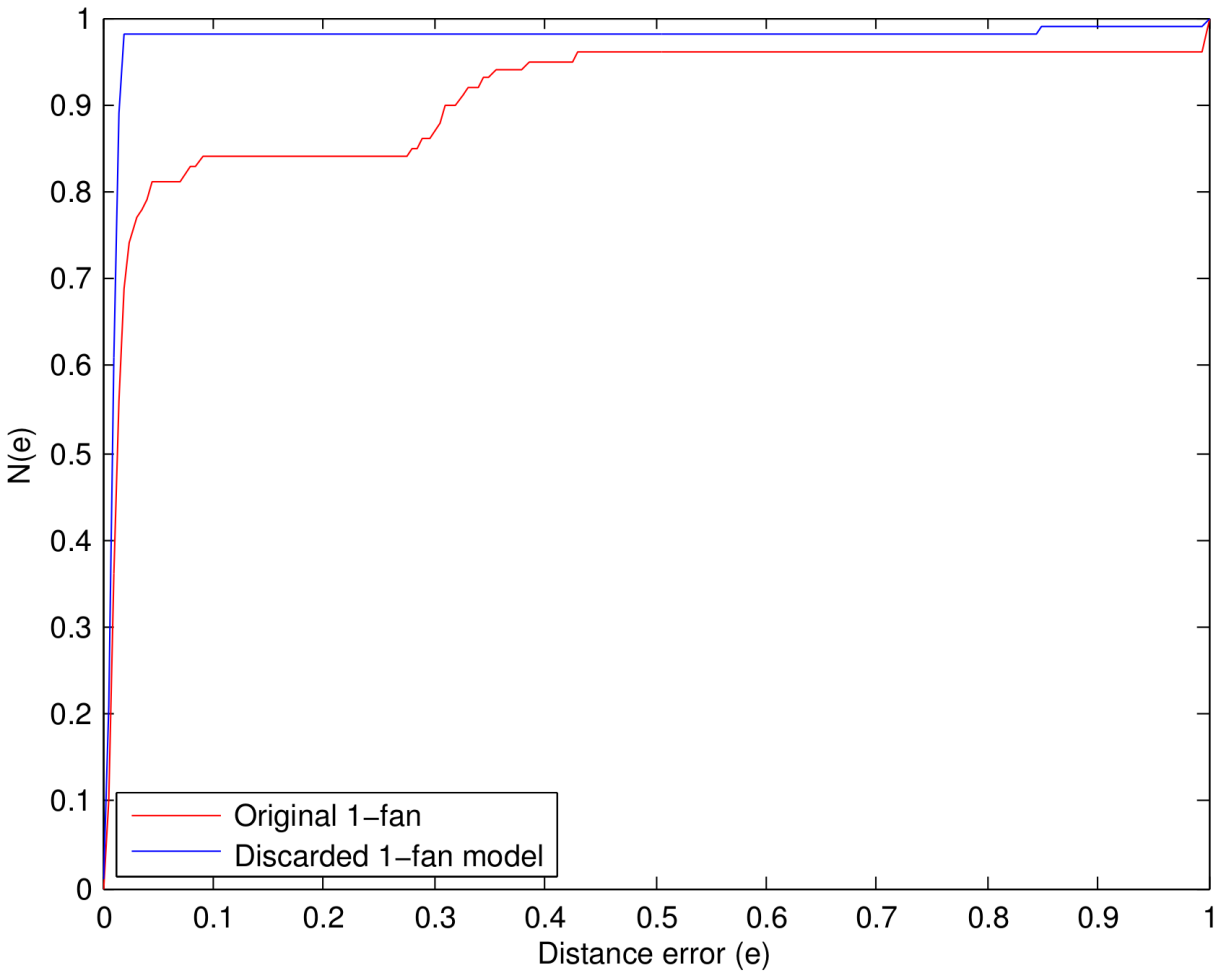
Influence of disabled parts on the Faces dataset.

We first demonstrated the influence of the disabled parts on the normal ones. We chose 200 images from the Faces dataset to train a 1-fan model, and chose 100 test images to construct a test dataset by shading the right eye and the right corner of the mouth in each test image. Then, we compared the discarded 1-fan model (the disabled parts had been discarded from the 1-fan model) (shown in Figure 6 (b)) with the original 1-fan model (shown in Figure 6 (a)). We used the distance error 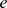 as the evaluation index. The distance error 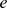 of all of the test images was normalized to 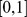. The comparison result is illustrated in Figure 7, where 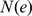 is the distribution function of the distance error. The horizontal axis represents normalized 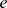, the vertical axis represents the percentage of test images whose distance error is smaller than 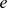. It is obvious that the higher the curve is, the better the model performs. It should be emphasized that only the normal parts were gathered to calculate the distance error 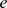. Figure 7 shows that the detection results of the discarded 1-fan were much better than that of the original 1-fan model, due to discarding the disabled parts. In other words, the disabled parts will severely affect the detection of the normal parts if they are not handled properly. Once the occlusion happens, the matching degree of the disabled parts is very likely to be low at the right positions, so they must search other positions to minimize the objective function, which would increase the deformation of the edge connecting them. As a result, the adjacent normal parts would tune their positions to reduce the edge cost (as illustrated in Figure 3.(c)). For this reason, we discarded the disabled parts in our method. The performance will be demonstrated in the following experiments.

**Figure 8:**
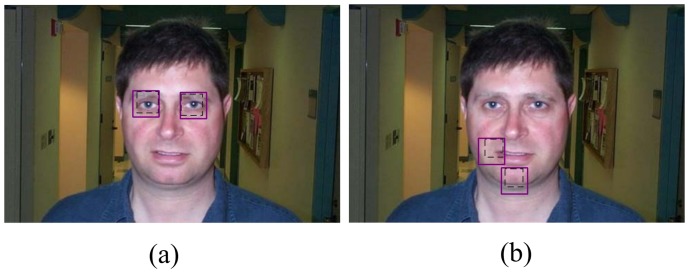
Sample images in the two-part-shaded test dataset. (a) is an image with part(1,2) being shaded (the occlusion degree is 81%). (b) is an image with part(4,6) being shaded (the occlusion degree is 64%). The purple solid boxes represent the part regions and the black dotted boxes reperesent the occlusion regions.

**Figure 9:**
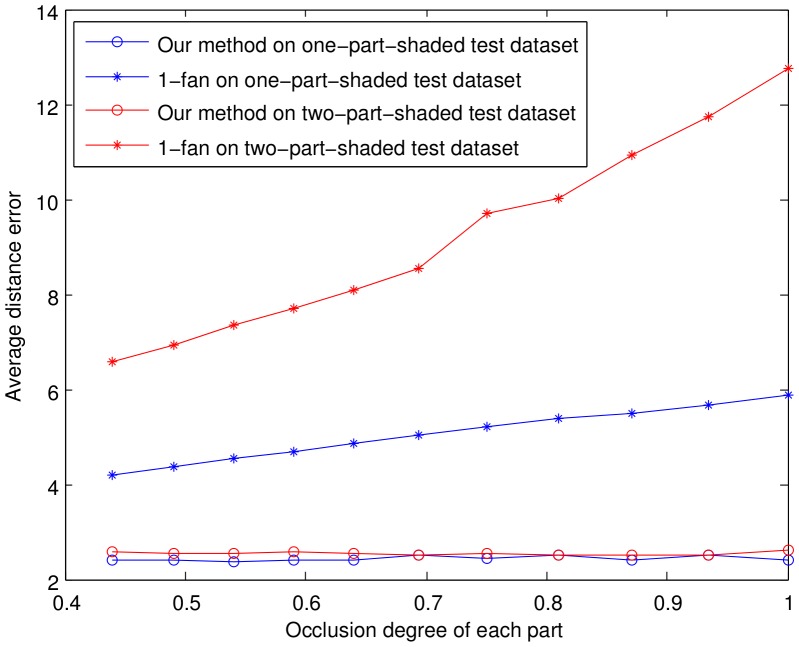
Average distance error of the partially occluded experiment.

In the second experiment (partially occluded experiment), we compared the proposed model with the 1-fan model and demonstrated the detection accuracy of our method when one or two parts were partially occluded. Both models were trained by 200 images selected randomly from the Faces dataset. We randomly selected another 100 images to construct the two test datasets. The first dataset, termed as the one-part-shaded test dataset, was constructed by shading part(1), part(2),…, part(6) respectively with different occlusion degrees for each test image. The occlusion degrees is defined as the ratio of the occlusion area to part area varied from about 44% (the size of the occlusion region was 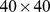) to 100% (the size of the occlusion region was 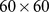), 11 values. The number of test images in the one-part-shaded dataset was 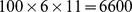. The second dataset, termed as the two-parts-shaded test dataset, was constructed by shading 7 kinds of adjacent part pairs (i.e., part(1,2), part(1,3), part(1,4), part(4,6), part(2,3), part(2,5), part(5,6)) respectively with different occlusion degrees for each test image. The number of images in the second dataset is 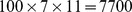. The test images in the two-parts-shaded test dataset are illustrated in Figure 8. The distance error 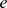 was also used as the evaluation index for detection accuracy. The average distance errors for all of the test images are plotted in Figure 9. As a typical instance, the results on the part(1,2)-shaded test are listed in Table 2.

**Table 2:** Average 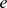 of the 1-fan model and our method when part(1,2) was shaded.

Occlusion degree of each part	Average 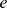 of 1-fan (pixel)	Average 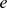 of Our model (pixel)
44.00%	6.6096	2.6243
49.00%	6.9762	2.6026
54.00%	7.3334	2.5952
59.00%	7.7257	2.6025
64.00%	8.1099	2.5939
69.44%	8.7296	2.5946
75.11%	11.6345	2.6098
81.00%	11.9330	2.6090
87.11%	14.0736	2.6091
93.44%	16.0132	2.6021
100.00%	18.3148	2.5845

Figure 9 shows that the average distance error for our model is almost constant and much smaller than that of the 1-fan model when the occlusion degree changes from 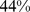 to 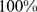. These results are due to the fact that the disabled parts were discarded from our model, and could not affect the detection of the normal parts. For the 1-fan model, the average distance error on the one-part-shaded dataset was smaller than that on the two-part-shaded dataset. What is more, the average distance error for the 1-fan model increase with the increase in the occlusion degree. Specifically from Table 2, once the occlusion degree exceeded 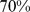, the average distance error increased sharply. That is because the information of the face held by part(1,2) (see Figure 8 (a)) was more than the other parts, thus the occlusion of part(1,2) greatly misguide the 1-fan model.

To further evaluate the performance of our method, we constructed four test datasets by complete shading one, two, three, or four parts (named the completely shading experiment). We carried out our algorithm on these test datasets. To evaluate the occlusion status variable 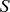, we used two evaluation indices: the occlusion false alarm probability 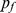 and the occlusion false dismissal probability 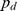 for all of the bits in the occlusion status variable 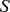. We defined the occlusion false alarm probability 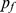 as the probability that the bit in 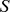 was wrongly estimated as 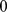, but it was actually 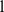. We defined the occlusion false dismissal probability 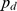 as the probability that the bit in 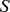 was wrongly estimated as 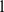, it was actually 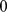. To evaluate the position variable 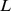, we also used the distance error 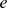 as the evaluation index. The results of complete shading experiment are shown in Table 3. We did not compare our method with the 1-fan model in this experiment because the 1-fan model almost cannot work in the case where three or more parts are shaded.

**Table 3:** The results of complete shading experiment on the Faces dataset.

Test set	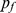	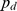	Average 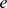 (pixel)
One-part-shaded test datasets	0.13%	0.67%	2.4057
Two-part-shaded test datasets	0.42%	0.50%	2.6009
Three-part-shaded test datasets	2.78%	1.11%	3.8830
Four-part-shaded test datasets	5.33%	1.83%	5.8602

From Table 3, we can see that both 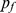 and 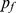 increase as the number of disable parts increases. That is because when more parts are occluded, it will be more difficult to obtain valid results in Step 1 of Algorithm 1 (Table 1), and the number of the valid two-part sub-models will be also reduced in Step 2 of Algorithm 1 (Table 1). Table 3 also shows that average distance error increases as the number of disabled parts increases. The reasons, except for those illustrated above, also lie in that the disabled parts are estimated only by the spatial relationship with the normal ones. The experimental results in Table 3 demonstrate that our method is competent for object detection even though most parts are occluded.

## Supporting Information

Appendix S1
**Deduction of** 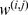 **.**
(PDF)

Figure S1
**Diagram of calculating** 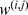 **.**
(TIF)
