# Supplementary material for: A Part-Based Probabilistic Model for Object Detection with Occlusion
Source: PLoS One. 2014 Jan 17;9(1):e84624. doi: 10.1371/journal.pone.0084624 (PMC3894947; doi:10.1371/journal.pone.0084624)
Supplement: Appendix S1 — Deduction of . (PDF) [file pone.0084624.s001.pdf]

Appendix

This section proves the expression about $w^{(i,j)}$ which is used in priori model of occlusion. $w^{(i,j)}$ represents the conditional probability of part v^j being shaded given the fact that v^i is shaded.

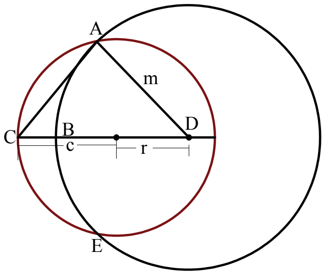

Figure 1. Diagram of calculating $w^{(i,j)}$.

Proof:

We assume that $c_o < d_{(i,j)} < 2c_o$, c_o is the radius of the circular occlusion, and the diagram is provided in Figure 1. For simpleness, let $c = c_o$, $m = d_{(i,j)}$. The brown circular is the occlusion circular, where v^i is located at point D, with a probability of $2\pi r \Delta r / \pi c^2$. Only when part v^j is located at Curve $\widehat{AE}$, the two parts will be shaded together, and this probability is $length\,of\,\widehat{AE}/2\pi m$, where $length\,of\,\widehat{AE}$ can be approximated by $2\sqrt{(c+r)^2 - m^2}$. So we have

$$
\begin{aligned}
w^{(i,j)} &= \int_{m-c}^{c} (2\pi r/\pi c^2) \cdot (2\sqrt{(c+r)^2 - m^2}/2\pi m) dr = \left[2 \int_{m-c}^{c} r\sqrt{(c+r)^2 - m^2} dr \right] /c^2\pi m \\
&= 2 \left(\int_{m-c}^{c} (r+c)\sqrt{(c+r)^2 - m^2} d(r+c) - c \int_{m-c}^{c} \sqrt{(c+r)^2 - m^2} d(r+c) \right) /c^2\pi m \\
&= 2 \left(0.5 \int_{m-c}^{c} \sqrt{(c+r)^2 - m^2} d(r+c)^2 - c \int_{m-c}^{c} \sqrt{(c+r)^2 - m^2} d(r+c) \right) /c^2\pi m \\
&= 2(\left[(c+r)^2 - m^2 \right]^{1.5} /3 \Big|_{m-c}^{c} \\
&-c \left[(r+c)\sqrt{(c+r)^2 - m^2} - m^2 \log\left((r+c) + \sqrt{(c+r)^2 - m^2} \right) \right] /2 \Big|_{m-c}^{c})/c^2\pi m \\
&= 2\left[4c^2 - m^2 \right]^{1.5} /3c^2\pi m - 2\sqrt{4c^2 - m^2}/\pi m + m \left[log((2c + \sqrt{4c^2 - m^2})/m) \right] /c\pi. \qquad (1)
\end{aligned}
$$
