# Supplementary figures and images for: A Part-Based Probabilistic Model for Object Detection with Occlusion
Source: PLoS One. 2014 Jan 17;9(1):e84624. doi: 10.1371/journal.pone.0084624 (PMC3894947; doi:10.1371/journal.pone.0084624)

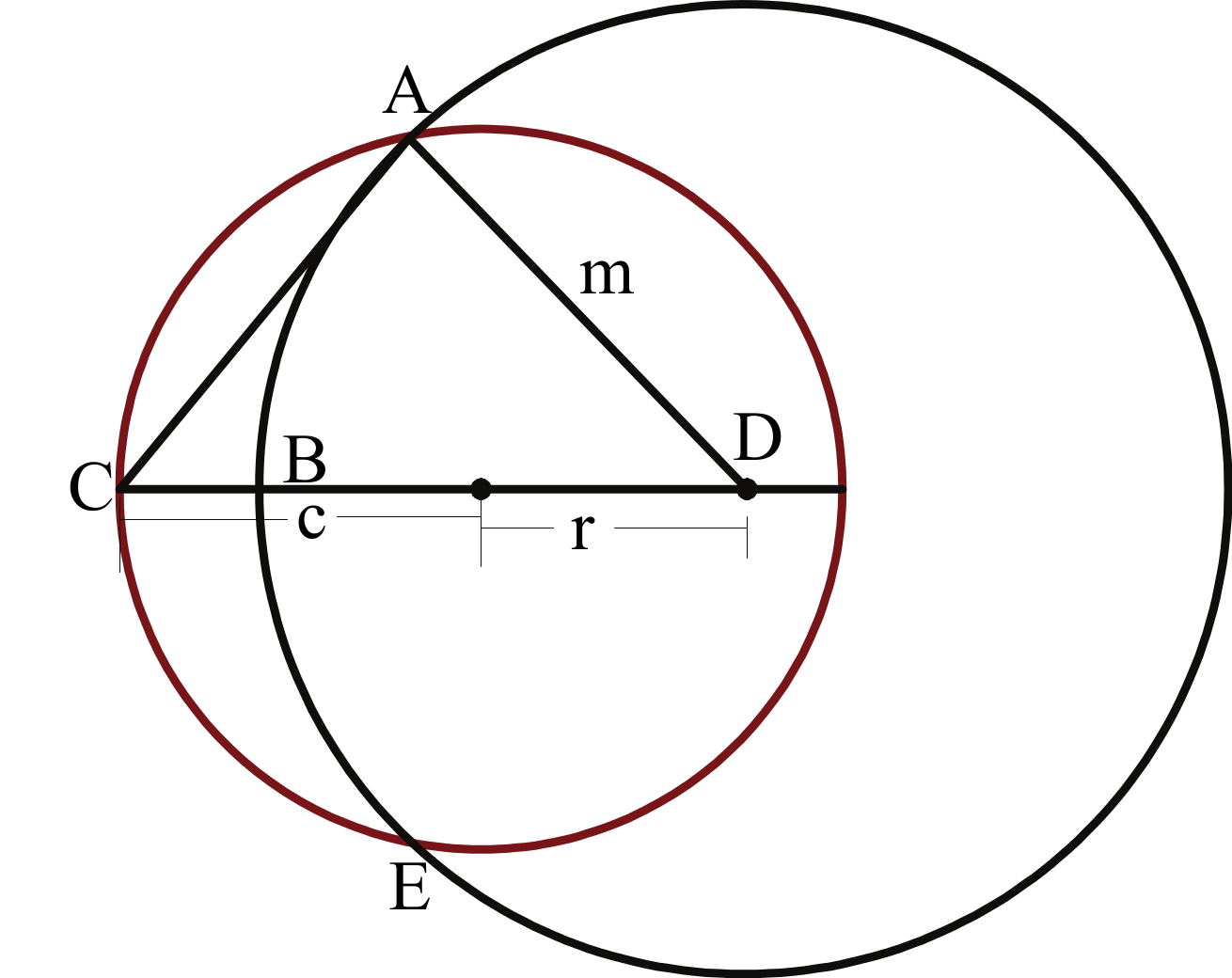

Supplement: Figure S1 — Diagram of calculating . (TIF) [file pone.0084624.s002.tif]
